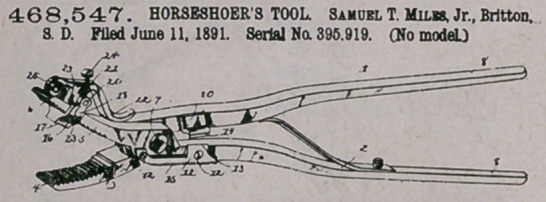# Recent Patents

**Published:** 1892-03

**Authors:** 


### RECENT PATENTS

RELATING TO

### VETERINARY MEDICINE AND ANIMAL INDUSTRY.

*Issued by U. S. Patent Office ending February, 1892.*

**464,558.** MARSH-HORSESHOE. JOSEPH D. DODGE, Rowley, Mass. Filed Sept. 5, 1890. Serial No. 364,004. (No model.)

*Claim.*—The combination, with a shoe A, of clamps hinged to the upper face of the shoe, one at each side, each clamp having reverse compound curves affording bearings upon the hoof at the bottom rear portion thereof upon each side and upon the forward or bridge portion thereof, said clamps being provided with downwardly-depending arms which are attached to the forward portion of the shoe, and also provided with adjustable clamping devices at the front, whereby they may be adjustably clamped, as set forth.

**468,230.** COLLAR-PAD. ADAM SCOTT, Wilmington, Ohio. Filed Apr. 21, 1890. Serial No. 348,754. (No model.)

*Claim.*—1. The combination, with a pad, of the plate 5 and the bridge 7, mounted on the pad and provided with the upper loop 10 and the lower series of loops 14, 15, and 16, substantially as described.

2. The combination, with a pad, of a curved plate secured thereto, an inverted-U-shaped bridge mounted on the plate and having its terminals connected by a lower transverse cross-bar, an open loop located upon the upper portion of the bridge, and a lower U-shaped-loop located upon the cross-bar and of a size to trisect the same and form opposite end loops, the upper loop being designed for the reception of the hame-connecting strap and the lower loops for either a single or double collar-connecting strap, substantially as specified.

3. The combination, with a pad, of the curved plate secured thereto and provided with a central opening registering with a similar opening in the pad, an inverted-U-shaped bridge straddling the opening formed in the pad and provided with a transverse cross-bar connecting the terminals above the opening, an open loop located upon the top of the bridge, and a lower loop located upon the cross-bar and forming a series of strap-passages or loops, substantially as specified.

**465,620.** HORSESHOE-CALK. GEORGE W. WEMPLE, Boston, Mass. Filed Jan. 15, 1891. Serial No. 377,830. (No model.)

*Claim.*—1. A horseshoe-calk provided with a plurality of shanks to enter holes in the body of the shoe for securing said calk thereto, the main body of which is composed of a hardened-steel wearing portion and a soft-iron back or upper portion, and the shanks made of soft iron and inserted through holes in said soft-iron back and the whole welded together, substantially as described.

2. In removable horseshoe-calks, the combination of the main body of the calk, composed of the hardened-steel wearing portion A and the soft-iron cap B, provided with the ribs *c c* and the holes *b b*, and the shanks *a a*, provided with flat heads *a'*, the parts being assembled together and firmly welded together, substantially as described.

3. A detachable horseshoe-calk comprising the tread or wearing portion A, having its upper portion made dovetailing in cross-section and made of steel, the soft-iron cap or upper portion B, provided with the lips *c c* and holes *b*, and the soft-iron shanks *a*, provided with the heads *a'*, said shanks being inserted through the holes *b* in the cap B with their heads between the ribs *c c*, and the dovetailed edge of the tread portion A being inserted between the ribs *c c* and in contact with the heads *a'*, and the whole compacted by applying pressure thereto while in a cold state and welded firmly together, substantially as described.

**466,884.** REMOVABLE HORSESHOE-CALK. SIMEON BUDLONG, Cumberland Hill, R. I. Filed Aug. 3, 1891. Serial No. 401,520. (No model.)

*Claim.*—1. As an improved article of manufacture, the auxiliary horseshoe hereinbefore described, the same consisting of the bent frame *b*, provided at the toe and heel with elongated openings extending transversely through the frame, sheet-metal calks fitting said openings and having the frame further provided with integrally-formed hooks *h*, arranged to receive the heel ends of the permanent shoe, and a movable key or wedge mounted in the toe portion of said frame, substantially as described, and for the purpose set forth.

2. The improved auxiliary horseshoe, consisting of the bent toe portion, having removable calks mounted therein and having a movable key or wedge arranged to engage with the permanent shoe, and further consisting of the two rearwardly-extending calk-holding heel portions arranged to be secured to the said toe portion and having the outer or free end of each heel provided with a hook *h* to receive the corresponding ends of the permanent shoe, substantially as hereinbefore described.

3. The combination, with a permanent horseshoe *a*, unprovided with calks, of the detachable auxiliary shoe *b*, the latter being provided with removable sheet-metal calks *c*, hooks *h*, having the rear ends of the permanent shoe held therein, and a movable wedge or key mounted in the toe portion of the shoe *b*, engaging the shoe *a* and locking the two together, substantially as hereinbefore described.

**464,773.** CURRY-COMB. Frederick E. Kohler, Canton, Ohio. Filed Apr. 22, 1891. Serial No. 389,884. (No model.)

*Claim.*—1. As an improved article of manufacture, a curry-comb composed of a series of rings serrated on one side concentric to each other, and a handle to extend over and embrace said rings, having at its end portions hooks B and C and lugs D and in its body portion kerfs a, b, and c, and the bolt B, by which the serrated rings are secured to the handle, substantially as described.

2. The combination, in a curry-comb, of a series of toothed rings concentric to each other and spaced apart, a bolt inserted through said rings to allow the same to rock thereon, and a handle secured to the ends of the bolt and spacing said rings and provided with kerfs into which the upper edge of the rings are located and retained spaced apart, substantially as set forth.

**465,674.** HORSESHOE. Joel Miller, Philadelphia, Pa. Filed Dec. 20, 1889. Serial No. 334,377. (No model.)

*Claim.*—The within-described horseshoe, having a grooved body with inner and outer flanges extending completely around the shoe, a rope filling contained within said groove and likewise extending completely around the shoe, a box or casing formed on the toe part of the shoe in advance of the outer flange of the grooved body, a hardened calk secured in said box or casing, and a flange extending around the top of the shoe and serving both to receive the nails and to brace the calk-receiving box at the toe of the shoe, substantially as specified.

**468,238.** BRIDLE-BLINDER. Robert G. Cather, Flemington, W. Va. Filed Aug. 10, 1891. Serial No. 402,269. (No model.)

*Claim.*—1. The combination, with a bridle or headstall, of the blinder consisting of two parts, each rigidly and directly secured to the forehead-strap and at the lower end adjustably secured to the cheek-strap and open at the side between the points of attachment and adapted to come directly in front of the horse's eyes, substantially as described.

2. In combination with a headstall or bridle, the blinder attachment, as described, rigidly secured at the top to the forehead-strap and at the lower ends adjustably secured to the cheek-straps and open at the sides between the two points of attachment and united at the central part, all substantially as described.

**468,252.** HORSESHOE. William H. Moore, Owen Sound, Canada. Filed Apr. 22, 1891. Serial No. 389,957. (No model.)

*Claim.*—1. A horseshoe round at the toe and having its toe-calk set near the center of the front of the shoe and at an angle pointing outwardly and backwardly, substantially as described.

2. A horseshoe round at the toe and having a toe-calk set near the center of the front thereof at an angle pointing outwardly and backwardly, the outside of the shoe being heavier than the inside and its heel flared outwardly and rearwardly and beyond the heel-calk on the inside of the shoe, substantially as described.

**468,299.** SAFETY-BRIDLE AND RELIEVABLE BIT. John Atkinson, New York, N. Y., assignor of one-third to Charles J. Mortimer, same place. Filed Sept. 9, 1890. Renewed Dec. 5, 1891. Serial No. 414,194. (No model.)

*Claim.*—1. A bridle having a spring shield or frame adapted to stride the nose, provided with eyes or loops for the rein, in combination with a bit composed of hinged, inwardly-curved, and movable prongs for the mouth of the horse, and means for compressing the shield for closing the nostrils and operating the prongs for clamping the jaw, substantially as herein set forth.

2. The bridle composed of the spring-strip extending down the face of the horse, adjustable at its upper end, and provided at its lower end with a shield or laterally-projecting wings curved around the face of the animal over the nostrils, in combination with the metallic bit-frame under the jaws, substantially as herein set forth.

3. A bridle having on the under side of the jaws a bar provided at each end with links hinged thereto and at the other ends of said links the hinged, upwardly-extending, and inwardly-curved prongs for the mouth of the animal, with the downwardly-projecting arms and adjustable springs connected therewith on the under side of the bar and with the outwardly-extending arm for attaching the rein thereto, substantially as herein set forth.

4. A bridle having on the under side of the jaw a vertically-disposed metal strip the upper end of which is in engagement with the throat-strap and the lower end being provided with a cross-bar, with links and upwardly-projecting inwardly-curved prongs hinged thereto, the downwardly-projecting arms and adjusting-springs, and the outwardly-extending arms for the reins, with the flexible shield on the nose of the animal, having the eyes or loops thereon, and the rein for controlling the movements of the shield and the prong-bit, substantially as herein set forth.

5. In a bridle, the within-described bit, constructed substantially as set forth, having the two prongs or fingers G, hinged or pivoted thereto, and provided with the arm P, and the socket Q, hinged thereto and adapted to be moved by the reins, whereby said fingers or prongs can be clamped upon the jaw.

**467,422.** NOSE-RINGING TOOL. CHARLES H. MARSHALL, Wood River, Nebr. Filed Mar. 26, 1891. Serial No. 386,506. (No model.)

*Claim.*—The nose-ringing device described and shown and adapted for use with the various forms of rings found in the market, said device comprising the pivoted elongated jaws A A′, each of which is formed with a concave inner face and each having therein a series of transverse arcuate grooves or depressions, each of said depressions being of different form and adapted to a different style of nose-ring, as herein shown and described.

**467,973.** CRIB FOR FEEDING CATTLE. EDWIN G. HASTINGS, Des Moines, Iowa. Filed Apr. 6, 1891. Serial No. 387,852. (No model.)

*Claim.*—1. In a crib for storing feed and feeding animals, a frame composed of posts at the corners and sides and straight pieces fixed together in crossed position and fixed to said posts at their lower ends on one side of the frame and to the posts on the other side of the frame at their top portions to project outward and upward, and platform-sections hinged at the top portions of said projections, arranged and combined to operate in the manner set forth, for the purposes stated.

2. An improved crib for feeding large and small animals, comprising a four-sided frame, in which the sides are connected by straight pieces fixed together in crossed position at their central parts and their upper ends projected outward and upward to support platform-sections and a roof, platform-sections hinged to the top ends of the said projections, floors fixed to the lower portions of said crossed pieces, a trough extending along the lower ends of the crossed pieces and floor thereon and outside of the side walls, openings in the said walls to allow feed to slide out into the said troughs, vertically-moving slides to close said openings in the wall, troughs extending inside of the side walls and under the troughs on the outside of the wall, openings between the upper and lower troughs to allow feed to fall from the upper into the lower troughs, and horizontally-moving slides to close the said openings, arranged and combined to operate in the manner set forth, for the purposes stated.

3. In a crib for feeding large and small animals, the vertically-sliding cut-off N and the horizontally-sliding cut-off M, in combination with a manger above the said cut-off M and a trough below the same cut-off, arranged and combined with the inclined floor and the side wall, to operate in the manner set forth, for the purposes stated.

**468,130.** HORSESHOE. MAU[illegible] D'AMILLY and JUSTIN CAILLET, Paris, France, assignors to William George Tiffany, Baltimore, Md. Filed Nov. 11, 1890. Serial No. 371,112. (No model.) Patented in France May 31, 1890. No. 206,024.

*Claim.*—The horseshoe-body *a*, made with the downwardly-diverging slot *c* and combined with the soft filling *b*, which fills and extends through said slot at top and bottom, all arranged so that the nails must be passed through said soft filling to secure the shoe to the hoof, as specified.

**468,205.** SLING-CIN[illegible]. DENIS O[illegible]LIVAN, Spokane Falls, Wash. Filed Dec. 31, 1890. Serial No. 376,398. (No model.)

*Claim.*—1. A sling-cinch composed of a strap having at one end a metal fastening consisting of a hook having a lateral outlet, an anti-friction roller *e* in the bend of the hook, and a cramping-pawl *g*, closing the outlet of the hook, substantially as shown and described.

2. A sling-cinch composed of a strap B, the plate *a*, fastened to said strap, the hook C, pivoted or hinged to the plate *a* and having an anti-friction roller in the bend, and a cramping-pawl in the mouth of

3. A sling-cinch composed of a strap having at one end a metal fastening composed of a hook having a lateral outlet, with an anti-friction roller located in the bend of the hook, and a cramping-pawl closing said outlet and having at the other end a plate or attachment provided with an offsetting eye, substantially as shown and described.

**468,547.** HORSESHOER'S TOOL. SAMUEL T. MILES, Jr., Britton, S. D. Filed June 11, 1891. Serial No. 395,919. (No model.)

*Claim.*—1. The herein-described tool, consisting of the opposite pivoted members terminating at their outer ends in jaws, the tooth-faced clinching-block mounted on one of the jaws, provided upon its upper side with a pair of perforated bearing-ears and at its opposite edges with teeth for engaging the teeth of the jaw, a V-shaped cam-lever having trunnions bearing in the ears, and a coiled spring pivoted at one end to the apex of the cam-lever in rear of its pivot and having its coiled end bearing on the jaw, substantially as specified.

2. The herein-described tool, consisting of the opposite members pivoted together, the cutting member 10, beveled to form a cutting-edge 14, extending laterally from one of the members, and the steel plate 11, secured to the side of the opposite member, beveled upon its back to form a cutting-edge 14 and at its lower edge toothed to form the rasp 15, substantially as specified.

## Figures and Tables

**Figure f1:**
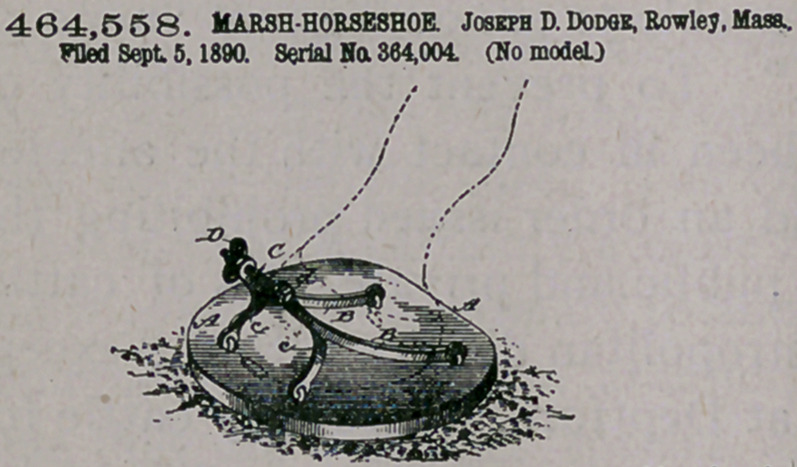


**Figure f2:**
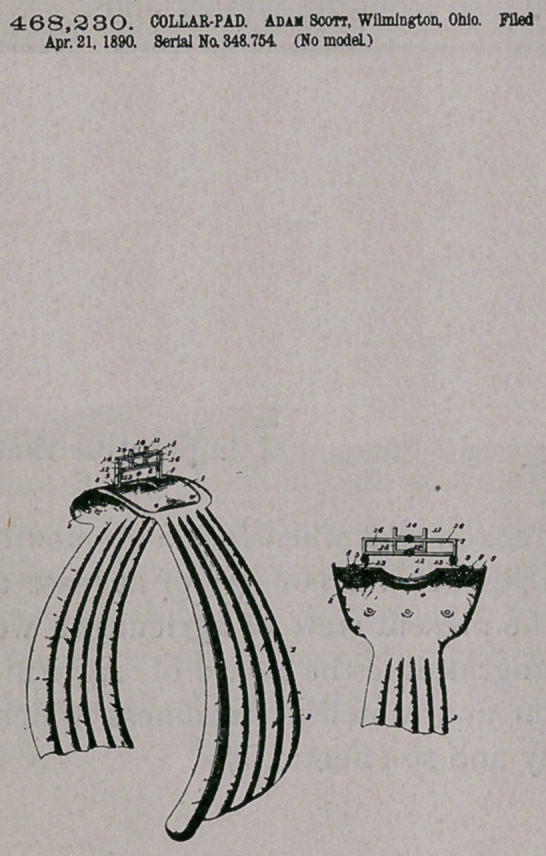


**Figure f3:**
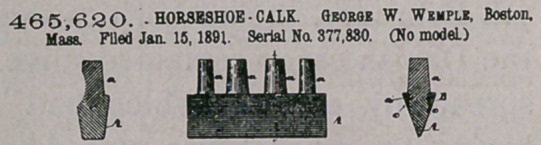


**Figure f4:**
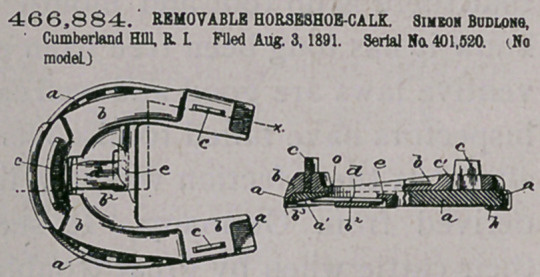


**Figure f5:**
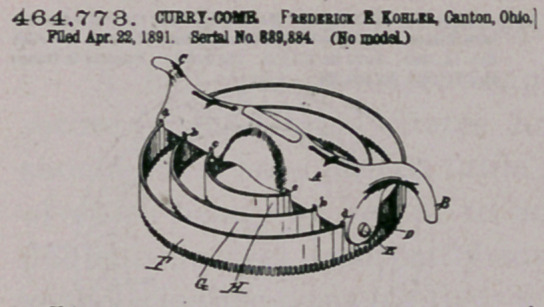


**Figure f6:**
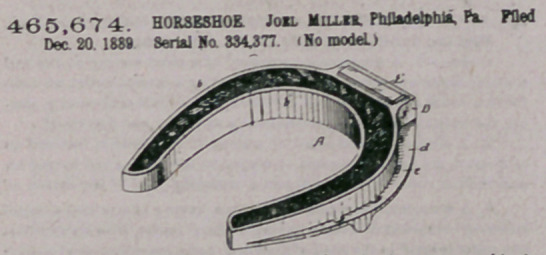


**Figure f7:**
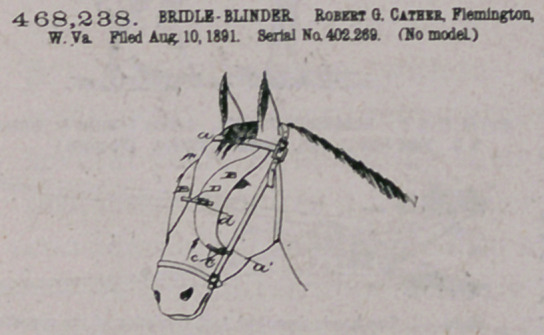


**Figure f8:**
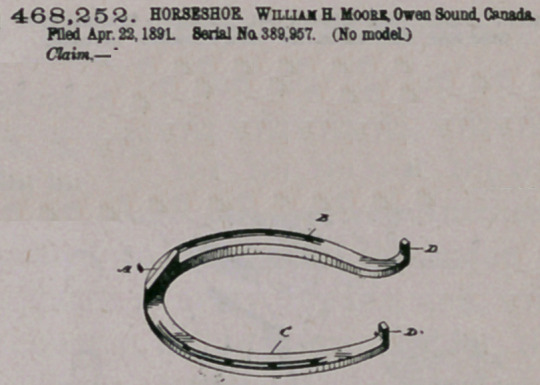


**Figure f9:**
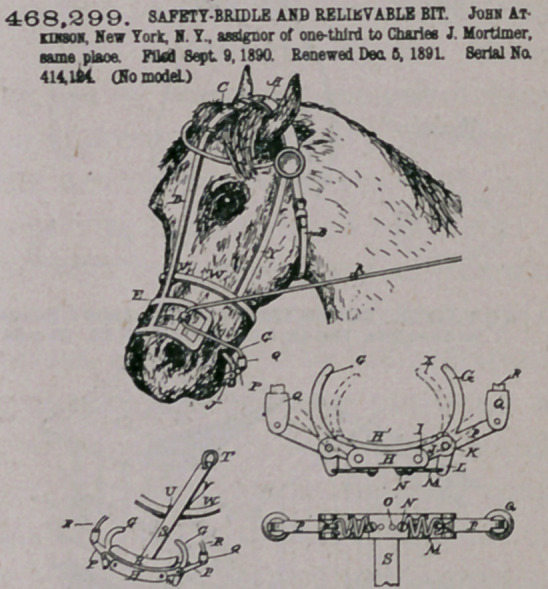


**Figure f10:**
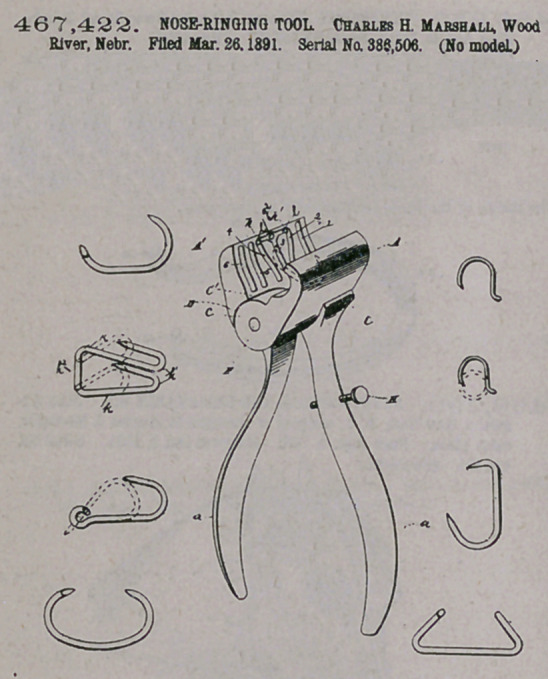


**Figure f11:**
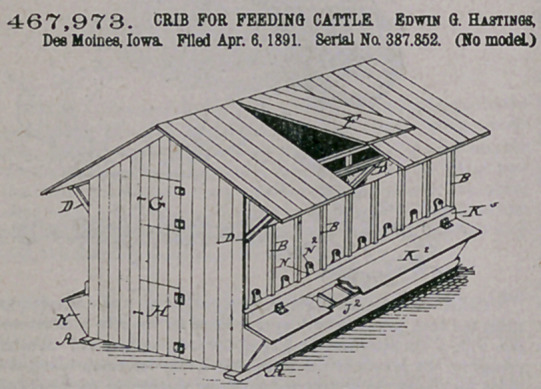


**Figure f12:**
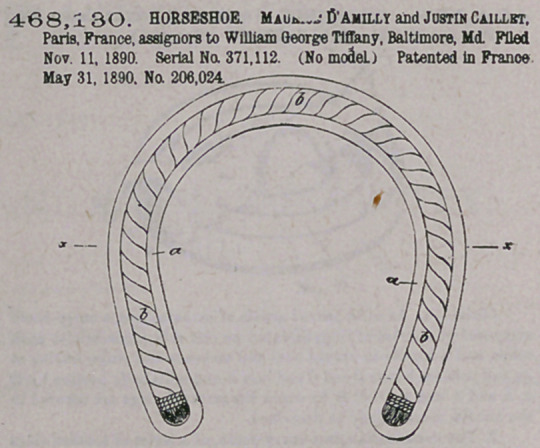


**Figure f13:**
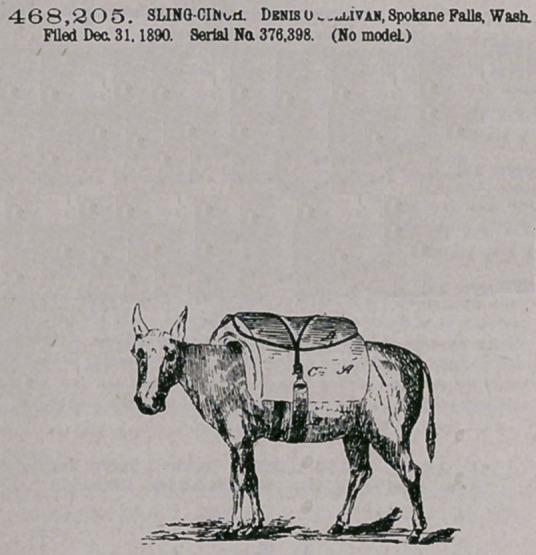


**Figure f14:**